# In Vitro Activity of *Allium cepa* Organosulfur Derivatives against Canine Multidrug-Resistant Strains of *Staphylococcus* spp. and *Enterobacteriaceae*

**DOI:** 10.3390/vetsci11010026

**Published:** 2024-01-09

**Authors:** Alba Maroto-Tello, Tania Ayllón, María Arántzazu Aguinaga-Casañas, Juan José Ariza, Silvia Penelo, Alberto Baños, Gustavo Ortiz-Díez

**Affiliations:** 1 Departamento de Microbiología, DMC Research Center, 18620 Granada, Spain; albamaroto@domca.com (A.M.-T.); arancha.aguinaga@domca.com (M.A.A.-C.); abarjona@dmcrc.com (A.B.); 2 Facultad de Ciencias de la Salud, Universidad Alfonso X el Sabio, 28691 Madrid, Spain; 3 Departamento de Genética, Fisiología y Microbiología, Facultad de Ciencias Biológicas, Universidad Complutense, 28040 Madrid, Spain; 4 Departamento de Microbiología, Campus Fuente Nueva, Universidad de Granada, 18001 Granada, Spain; jariza@dmcrc.com; 5 Servicio de Urgencias, Hospitalización y UCI, Hospital Clínico Veterinario Complutense, Universidad Complutense, 28040 Madrid, Spain; 6 Departamento de Medicina y Cirugía, Facultad de Veterinaria, Universidad Complutense, 28040 Madrid, Spain; gusortiz@ucm.es

**Keywords:** antibiotic resistance, *Allium* extracts, dogs, multidrug-resistant *Enterobacteriaceae*, multidrug-resistant *Staphylococcus* spp.

## Abstract

**Simple Summary:**

The rise of drug-resistant bacteria, particularly in animals, poses a major challenge in veterinary medicine. Antibiotic development lags behind the increasing resistance. To tackle this, alternative therapies have been explored, such as the use of natural products and plant extracts. This study evaluates the laboratory efficacy of plant derivatives of the *Alliaceae* group (which includes garlic and onion) as antimicrobial agents, with encouraging results. Although further research is needed, these findings suggest a potential role for these natural compounds in veterinary medicine.

**Abstract:**

Background: The increase of multi-resistant bacteria, especially *Staphylococcus* spp. and *Enterobacteriaceae*, constitutes a challenge in veterinary medicine. The rapid growth of resistance is outpacing antibiotic discovery. Innovative strategies are needed, including the use of natural products like *Allium* species (*Allium sativum* L. and *Allium cepa* L.), which have been used empirically for centuries to treat infectious diseases in humans and farm and aquaculture animals due to their antibacterial properties. Methods: This study aimed to evaluate the in vitro activity of two *Allium*-derived compounds, propyl propane thiosulfinate (PTS) and propyl propane thiosulfonate (PTSO), against multi-resistant *Staphylococcus* spp. (*n* = 30) and *Enterobacteriaceae* (*n* = 26) isolated from dogs referred to a veterinary teaching hospital in Madrid. Results and Discussion: The results indicated the in vitro efficacy of PTSO/PTS against the tested bacterial strains, and 56.7% of *Staphylococcus pseudintermedius* and 53.8% of *Enterobacteriaceae* showed sensitivity to PTS and PTSO compared with classic antibiotics. In addition, 50% of *S. pseudintermedius* strains resistant to erythromycin, ibofloxacin, difloxacin and orbifloxacin and 50% of *Enterobacteriaceae* strains resistant to tetracycline and doxycycline were sensitive to PTS and PTSO. Although studies are needed to verify their efficacy in vivo, the combined use of PTS and PTSO exhibits promise in enhancing bacterial sensitivity against *S. pseudintermedius* and *Enterobacteriaceae* infections, providing a first insight into the potential of both compounds in veterinary practice.

## 1. Introduction

The World Health Organization (WHO) recognizes antimicrobial resistance as one of the most important issues affecting public, animal and environmental health [1]. The proximity between dogs and humans entails a potential risk of pathogen transmission, including antibiotic-resistant bacteria [2]. Canines can transmit many pathogens to humans, causing infections ranging from skin rashes to life-threatening bacteremia [3].

Antibiotics are critical for combating infectious diseases [4]. However, their excessive and inappropriate use combined with inadequate waste management and spread to the environment has contributed to the development of antibiotic-resistant strains and increased mortality due to infectious diseases [5]. Recent studies have shown that Gram-negative bacteria often exhibit multidrug resistance (MDR), including to critically important antimicrobials (CIAs), highlighting the complex challenge they pose in antimicrobial resistance [6,7]. Primary Gram-negative bacteria with zoonotic potential that cause healthcare complications, such as nosocomial, urinary tract and bloodstream infections, belong to the genus *Enterobacteriaceae*, which includes *Escherichia coli*, *Klebsiella* spp. and *Enterobacter* spp. Among Gram-positive bacteria, methicillin-resistant *Staphylococcus* spp. (MRS) and vancomycin-resistant *Enterococcus* spp. (VRE) are of particular concern [8,9].

Resistance outpaces the rate at which new antibiotics are discovered, making antibiotics a finite healthcare resource [10]. As a result, we are currently faced with a situation where the therapeutic options for infections are limited because certain pathogenic strains are resistant to all existing groups of antibiotics [11]. Addressing this challenge requires urgently exploring new antimicrobials, developing additional agents and investigating innovative chemical structures for enhanced efficacy. These efforts are crucial not only for curing existing infections but also for reducing the risk of future infections in both animals and humans. An integral part of this approach includes controlling bacterial growth in animal feed, which is a key factor in preventing zoonosis and may involve the use of specialized feed additives [12,13,14].

Medicinal plants are rich sources of novel compounds with potential antimicrobial properties [15]. However, few veterinary medicine studies have evaluated the efficacy of these plant extracts against antibiotic-resistant bacteria [16,17]. Among different medicinal plants, *Allium* species have been used worldwide for centuries to treat infectious diseases. A substantial amount of historical evidence is available, including documents dating back more than 3500 years from ancient Egypt describing the medicinal properties of this genus [18,19,20]. In recent years, the antibacterial properties of *Allium* plant extracts have been extensively studied, including their efficacy against multidrug-resistant bacteria [21,22].

One of the most well-known components of this botanical family is diallyl thiosulfinate (allicin), which is present in *Allium sativum* L., and its antimicrobial activity has been widely reported [23,24,25,26]. However, its instability limits its suitability as a therapeutic agent in pet food. Allicin degrades easily to other organosulfur compounds such as ajoenes, vinyl dithiins and diallyl polysulfides (DAPS) at 20 °C [27,28,29,30]. The proportion of these degradation products can vary depending on the *Allium sativum* L. processing conditions. In addition, allicin degradation in DAPS raises safety concerns because high concentrations of these molecules with multiple sulfur atoms have a higher potential to oxidize canine erythrocytes than non-degraded thiosulfonates [30]. This could explain the conflicting results regarding the safety of *Allium sativum* L. in pet diets, with a few studies considering it harmful [31]. However, others report improved animal health with dietary supplementation [32,33].

In contrast, the organosulfur compounds present in *Allium cepa* L., propyl propane thiosulfinate (PTS) and propyl propane thiosulfonate (PTSO), have been reported to be safe and not cause toxicity [34,35,36]. These molecules exhibit greater stability because, although PTS leads to propyl disulfide (PDS) and PTSO through a disproportion reaction, PDS can be transformed into PTSO in the presence of oxygen [37]. PTS and PTSO have demonstrated significant antimicrobial activity in vitro and in vivo against different pathogenic strains in livestock, including *Staphylococcus* spp., *Enterobacteriaceae* spp. and *Enterococcus* spp. [38,39,40]. In addition, PTS and PTSO have shown in vitro antimicrobial activity against multidrug-resistant bacteria and yeasts isolated from human clinical samples [41]. However, information on the antimicrobial activity of these compounds against antibiotic-resistant bacteria, which commonly affect dogs, is lacking. Methicillin-resistant *S. pseudintermedius* (MRSP) is an important pathogen commonly encountered in canine infections. Due to its genetic similarities to *S. aureus*, MRSP poses a considerable concern for veterinary medicine and public health, as it can be transmitted between animals and humans. *Enterobacteriaceae* strains such as *K. pneumoniae* and *E. coli* are among the most common bacterial pathogens observed in dogs [42].

This study evaluated the in vitro antimicrobial activity of the *Allium*-derived compounds PTS and PTSO, compared to commonly used antibiotics, against different strains of multi-resistant *Staphylococcus* spp. and *Enterobacteriaceae* isolated from dogs.

## 2. Materials and Methods

### 2.1. Study Design and Setting

In this study, a total of 56 multi-resistant bacterial strains obtained as etiological agents from hospitalized dogs in a previous study performed at Alfonso X El Sabio University in Madrid were selected [2]. Of these, 30 strains, identified as S. *pseudintermedius*, showed the presence of the *mec*A gene, which confers resistance by producing a penicillin-binding protein PBP 2A, and 26 species of *Enterobacteriaceae* [43] (19 *E. coli* and 7 K. *pneumoniae*) had at least one extended-spectrum beta-lactamase resistance gene (*bla*_CTX-M1_, *bla*_CTX-M9_, *bla*_SHV,_ *bla*_TEM_). *E. coli* CECT 516 and *S. pseudintermedius* DSM 21284 were used as reference strains. Samples were stored at −20 °C at Alfonso X El Sabio Veterinary Hospital (Madrid, Spain).

### 2.2. Sensitivity of the Selected Strains to Antibiotics and Allium-Derived Compounds

The antimicrobial susceptibility of *Staphylococcus pseudintermedius* and *Enterobacteriaceae* strains was tested in an external laboratory (Laboklin, Madrid, Spain) using a microdilution test and microtiter plates (Micronaut S Kleintiere, Merlin Diagnostika GmbH, Bornheim-Hersel, Germany). Resistance patterns were determined using a set of standardized antimicrobials, following recommendations of the National Committee for Clinical Laboratory Standards (CLSI) [44] guidelines and clinical breakpoints established by the European Committee of Antimicrobial Susceptibility Testing (EUCAST), including enrofloxacin, marbofloxacin, orbifloxacin, difloxacin, ibafloxacin, pradofloxacin, gentamicin, neomycin, kanamycin, tobramycin, sulfamethoxazole-trimethoprim, doxycycline, tetracycline, lincomycin, clindamycin, spiramycin-trimethoprim, erythromycin, fusidic acid, chloramphenicol, colistin, nitrofurantoin, rifampicin, streptomycin-trimethoprim, penicillin G, ampicillin, amoxicillin, amoxicillin-clavulanic acid, cephalexin, cefotixin, cefquinome, cefoperazone and cefovecin. All antibiotics were purchased from Sigma-Aldrich (Madrid, Spain) and dissolved according to the manufacturer’s recommendations. Erythromycin, clindamycin, fusidic acid and rifampicin were not tested against Gram-negative bacteria. Moreover, colistin has not been tested against Gram-positive bacteria.

PTS and PTSO from *Allium cepa* L. were supplied with high purity (97%) by Panadog-Enzim-Orbita (Tavira, Portugal) and dissolved in polysorbate-80 to a final concentration of 500 g/L.

To evaluate the antibiotic sensitivity of *Allium* compounds, the Minimum Inhibitory Concentration (MIC) was selected according to CLSI guidelines [44]. For this purpose, decreasing concentrations of the antimicrobial agents (2000—0.48 µg/mL) were prepared in 1:2 dilutions in wells of microtiter plates in Mueller–Hinton medium buffer (Scharlab, Barcelona, Spain) with a bacterial inoculum of the different strains in the logarithmic growth phase (approx. 10^5^ CFU/mL). In addition, uninoculated broth was used as a negative control, and another well with only a bacterial suspension (without antibiotics) was used as a positive control. All samples were performed in duplicate. Samples were incubated in a tube rotator (VWR; Barcelona, Spain) for 24 h at 20 rpm. Subsequently, they were measured using a Multiskan FC Microplate Reader Spectrophotometer (Thermo Scientific, Waltham, MA, USA) at a wavelength of 620 nm. The microorganism was expected to grow in the control tube and in tubes that did not contain sufficient antimicrobials to inhibit its development. Subsequently, according to the methodology described by Turnidge and Paterson (2007) [45], the probability of clinical success for each compound was predicted by establishing a cut-off point of 62.5 µg/mL. Consequently, MICs above this value were considered resistant, whereas those equal to or lower than this value were considered sensitive.

### 2.3. Statistical Analysis

Data analysis was performed using IBM SPSS Statistics v19 software. The percentage of observed bacterial resistance was described along with the frequency distribution. McNemar’s test was performed to evaluate the association between resistance to *Alliaceae* compounds and the different antimicrobials tested. Statistical significance was set at *p* < 0.05.

## 3. Results

### 3.1. Staphylococcus pseudintermedius

The number and percentages of resistant strains obtained from the 30 previously selected antibiotic-resistant *Staphylococcus pseudintermedius* strains tested with PTS and PTSO, as well as with various antibiotics, are detailed in Table 1.

The reference strain *S. pseudintermedius* DSM 21284 had an MIC value of 31.25 µg/mL for PTS/PTSO, which provides a baseline of the activity of these *Allium cepa*-derived compounds. The highest resistance rates were observed against erythromycin (90.0%), ibafloxacin, difloxacin, enrofloxacin and orbifloxacin (83.3% each). In addition, among all multi-resistant strains, 43.3% of the evaluated S. *pseudintermedius* showed resistance to both PTS and PTSO. Despite observed variations in MIC values for PTS and PTSO, with two of the studied strains showing higher values for PTSO compared to PTS, the sensitivity results are still comparable for both compounds when considering the defined cut-off points.

The sensitivity to *Alliaceae* compounds and various antimicrobials was compared, revealing that a larger proportion of strains were sensitive to PTS and PTSO, as determined by the MIC cut-off of 62.5 µg/mL, in comparison to other tested antimicrobials. In contrast, resistance was observed to the following antibiotics: erythromycin, clindamycin, lincomycin, ibafloxacin, difloxacin, enrofloxacin, marbofloxacin, orbifloxacin, sulfamethoxazole-trimethoprim, streptomycin-trimethoprim and spiramycin-trimethoprim. All *Staphylococcus* strains containing the *mec*A gene were considered resistant to amoxicillin, ampicillin, cephalexin, cefotixin, cefquinome and cefoperazone. No nitrofurantoin-resistant strains were detected in this study. In addition, there was a higher proportion of tested strains that showed sensitivity to both *Allium*-derived compounds and resistance to erythromycin, clindamycin, ibafloxacin, difloxacin, enrofloxacin, marbofloxacin, orbifloxacin, chloramphenicol and trimethoprim/sulfamethoxazole, showing the highest percentage (50%) of the *S. pseudintermedius* strains resistant to erythromycin, ibafloxacin, enrofloxacin and orbifloxacin (Table 2; Supplementary Material: Tables S1 and S2).

### 3.2. Enterobacteriaceae

*Enterobacteriaceae* strains in the study exhibited different percentages of resistance to antibiotics, PTS and PTSO. The reference strain *E. coli* CECT 516 showed an MIC for PTS/PTSO of 62.5 µg/mL, which establishes a comparative baseline for the activity of these Allium cepa-derived compounds against Enterobacteriaceae. The highest resistance percentages were observed for tetracycline and doxycycline (96.2%), followed by ibafloxacin and difloxacin (88.5% each) and enrofloxacin, marbofloxacin and orbifloxacin (84.6% each), as shown in Table 3.

Additionally, 46.2% of the *Enterobacteriaceae* strains were resistant to PTS and PTSO. A statistically significant difference was observed in the sensitivity of PTS and PTSO compared with that of ibafloxacin, difloxacin, enrofloxacin, marbofloxacin, orbifloxacin, tetracycline and doxycycline. Due to intrinsic extended-spectrum beta-lactamase resistance genes, these strains have been classified as resistant to amoxicillin, ampicillin, cephalexin, cefotixin, cefquinome and cefoperazone.

Finally, *Enterobacteriaceae* strains demonstrated significantly lower resistance to kanamycin and neomycin than to PTS and PTSO. A higher proportion of the tested strains showed sensitivity to PTS/PTSO and resistance to neomycin, kanamycin, ibafloxacin, difloxacin, enrofloxacin, marbofloxacin, orbifloxacin, tetracycline and doxycycline, with the highest percentage (50%) of *Enterobacteriaceae* strains resistant to tetracycline and doxycycline. The results are detailed in Table 4.

Finally, when comparing the higher percentages (50%) of strains resistant to antibiotics and sensitive to PTS/PTSO (Table 2 and Table 4), a higher proportion of antibiotic-resistant *S. pseudintermedius* strains was observed than that for *Enterobacteriaceae*, indicating greater sensitivity to PTS/PTSO.

## 4. Discussion

Antibiotic overuse has led to the development of multidrug-resistant bacterial strains, making it increasingly challenging to treat infections in humans and animals [46,47]. Therefore, it is important to explore alternative therapeutic options. Natural products have emerged as valuable novel antimicrobial agent sources due to their diverse chemical compositions and potential therapeutic properties [48,49]. *Allium* spp., including *Allium cepa*, have been extensively studied for their medicinal properties, including antimicrobial activity [27,50,51]. Organosulfur compounds observed in *Allium cepa* L. and *Allium sativum* L. have shown promising antibacterial effects, making them potential candidates for use against multidrug-resistant bacteria [52,53].

Despite the considerable attention natural products have received in human medicine, their application in veterinary medicine remains largely unexplored. Limited studies have assessed the efficacy of natural products against canine bacterial strains. This lack of research highlights the need for further studies investigating the potential therapeutic uses of natural compounds in veterinary applications.

In our study, the combination of conventional antimicrobials with PTS and PTSO has demonstrated promising results in increasing the sensitivity of bacterial strains against infections caused by *S. pseudintermedius* and *Enterobacteriaceae*. This approach, as highlighted in our findings, suggests a significant alternative to traditional synergy, aligning with the guidelines for antimicrobial therapy combination [54] and underscoring the need for innovative strategies in the face of rising antibiotic resistance. The in vitro antibacterial activity of PTS and PTSO, as compared with other antimicrobials, has been shown to be effective against multidrug-resistant bacteria [41], supporting the potential of this combination. Furthermore, the importance of systematic mapping of the long-term clearance efficacy of drug combinations, as discussed in recent research [55], is crucial for designing more effective multidrug regimes, especially against persistent infections.

Canine-originating bacterial strains, such as multidrug-resistant *Staphylococcus* spp. and *Enterobacteriaceae*, are of great concern to both veterinary and public health [56,57]. These strains exhibit substantial diversity and can cause various infections in dogs and other domestic animals, with potential zoonotic implications for humans [58,59,60,61]. Furthermore, the ability of these strains to develop multi-drug resistance impairs treatment. Therefore, research on canine-specific bacterial strains is crucial for developing effective therapeutic strategies [62].

Several veterinary medicine studies have investigated the antimicrobial activities of natural products against canine *Staphylococcus* and *Enterobacteriaceae* spp. These studies explored a variety of natural sources, including herbal extracts, honey products and bacteriophages, among others [63,64,65]. These investigations provided valuable insights into the potential efficacy of natural compounds as alternative therapies, with botanical products being the most promising solutions, such as *Garcinia mangostana* or *Harungana madagascariensis* extracts [66,67]. Organosulfur derivatives from garlic, such as allicin-inspired compounds, have been reported to exhibit antibacterial activities against *Staphylococcus* spp. [68]. Similarly, in previous studies, PTS and PTSO from onions showed antimicrobial activity against *Staphylococcus* and *Enterobacteriaceae* multidrug-resistant strains isolated from human samples [41]. However, to our knowledge, this is the first study to evaluate the in vitro activity of these compounds against multidrug-resistant canine strains.

*S. pseudintermedius* is commonly isolated from superficial and deep pyoderma, otitis externa, urinary tract infections and other canine tissues [58,69,70,71,72], and it does not normally colonize humans, although transfer between owners and their pets has been described and, in certain cases, has been associated with pathologies [73,74,75,76,77]. Additionally, many *Staphylococcus* spp. carry the *mecA* gene, which encodes a penicillin-binding protein (PBP2a) that confers resistance to beta-lactams [78,79,80]. Recently, the prevalence of methicillin-resistant *Staphylococcus* (MRS) has increased [79]. MRS can express resistance to any combination of antibiotics, including aminoglycosides, fluoroquinolones, lincosamides, macrolides, tetracyclines, potentiated sulphonamides and rifampicin [72].

Based on the results of this study, it can be hypothesized that PTS/PTSO can effectively treat a higher percentage of methicillin-resistant *S. pseudintermedius* infections than beta-lactams. Furthermore, the tested strains showed lower resistance to PTS and PTSO than to most quinolones, which should be prescribed as a last resort. Additionally, as previously reported, MRS acquires resistance to fluroquinolones [81]. Most publications show similar results regarding resistance to these antibiotics and consider these drugs not to be good therapeutic alternatives for MRS treatment [71,82].

This study observed that PTS and PTSO did not show lower resistance rates than aminoglycosides, tetracyclines, rifampicin, chloramphenicol or fusidic acid. Regarding the susceptibility of *S. pseudintermedius* strains to the different antibiotics tested, our results agree with those obtained by other authors [83]. Although moderate-to-high resistance patterns have been described for aminoglycosides, their clinical efficacy as single agents against infections caused by *Staphylococcus* strains is not well established [84,85,86]. Rifampicin, an antibiotic developed in the past, has been of recent interest due to its activity against MRS. The moderate resistance described in this study is consistent with other publications [69,87,88,89,90,91], although resistant strains have been described [92]. The tetracycline resistance rates observed in this study are consistent with those reported in other studies [90]. Tetracyclines are more effective in vitro than in vivo against different species of *Staphylococcus* [88]. Fusidic acid resistance in this study was higher than that reported in other publications [65,93]. However, more studies are required to determine the correlation between in vitro studies and clinical efficacy. Chloramphenicol was used decades ago; however, it is not now widely used because of its narrow safety margin, the need for frequent administration and the lack of presentations suitable for small animals in most countries [88]. Nitrofurantoin did not induce in vitro resistance against any of the *Staphylococcus* isolates tested in this study. High susceptibility to this drug has been reported in other studies [94]. However, this drug is highly toxic and not used in clinical practice [95].

The global problem of antimicrobial resistance includes the emergence of extended-spectrum beta-lactamase (ESBL)-producing *Enterobacteriaceae* that cause considerable morbidity and mortality, especially *E. coli* and *K. pneumoniae* species [96]. The prevalence of canine isolates resistant to beta-lactams, including broad-spectrum cephalosporins, has increased in recent years [97,98], leading to urinary infections. Companion animals that live in close contact with humans may contribute substantially to their owners’ exposure to ESBL-producing *Enterobacteriaceae* [98].

In this study, all ESBL-producing *Enterobacteriaceae* isolates were found to be multidrug-resistant. The resistance percentages of ESBL-producing strains to PTS and PTSO were similar to those observed for amoxicillin-clavulanate, with a low resistance to amoxicillin-clavulanic acid, consistent with recent studies [99]. However, other authors reported higher values [100]. Bacteria with ESBL genes are associated with resistance to other non-beta-lactam antimicrobials, such as tetracyclines, quinolones, lincosamides, macrolides and, to a lesser extent, chloramphenicol and aminoglycosides [98,101,102,103,104,105,106]. Moreover, PTS and PTSO showed higher sensitivity percentages for ESBL strains than most quinolones and tetracyclines. The sensitivity percentages for these groups of antibiotics were similar to those observed in other studies [101,104,105,106] and do not usually represent a good therapeutic alternative. As observed in other surveys, aminoglycosides presented low resistance percentages, probably because of their limited use in the clinic due to their pharmacokinetics and potential side effects [107,108]. *Allium* compounds showed similar resistance to colistin, nitrofurantoin, chloramphenicol and potentiated sulfonamides in the ESBL strains. Colistin was discontinued because of nephrotoxicity. However, the emergence of carbapenem resistance in clinically important bacteria such as *Pseudomonas aeruginosa, Acinetobacter baumannii*, *K. pneumoniae* and *E. coli* has propitiated its reintroduction into clinical practice as a last-resort treatment option [109]. Resistance to nitrofurantoin has been described in low percentages of ESBL-producing *Enterobacteriaceae* [95]; however, its toxicity and poor pharmacokinetic characteristics have led to its low use in clinical practice. Chloramphenicol and potentiated sulfonamides have shown low to moderate resistance rates in recent publications [100], probably due to lower antibiotic pressure.

Finally, considering the historically safe use of PTS and PTSO in farm animal species and their dietary origin, both compounds are presumed safe for dog health. The toxicological aspects of PTS and PTSO have previously been tested in experimental animals. In vivo studies have demonstrated the low acute and subchronic oral toxicity of PTSO [34,35,36]. In addition, recent studies on rats orally administered with PTS and PTSO for 90 days showed no toxic effects [35]. Some studies found in the scientific literature show contradictory results about the safety of alliaceous derivatives in the canine diet, even considering them to be harmful food [31]. This fact largely contradicts the results of trials conducted by other authors in which the incorporation of alliaceous in the diet of dogs leads to an improvement in animal health [32,110]. For example, the trial conducted by Yamato et al. [33] involved orally administering a daily dose of garlic extract at 90 mg/kg of weight to Beagle dogs for 12 weeks, which not only resulted in the absence of adverse effects, but also improved the health of the animals by increasing the gene expression of antioxidant enzymes.

While PTS/PTSO is currently being commercialized for use in farm animals, such as pigs and poultry, research into its application in pets is a more recent development. This paper serves as a pioneering contribution in this field, exploring the use of PTS/PTSO in pet nutrition, and specifically for dogs. This study aims to provide foundational knowledge and encourage further research. Our findings contribute to an increase in the knowledge of this area, highlighting the need for continued research into the safe and effective use of PTS/PTSO in canine diets. Despite these promising results, further pharmacokinetic studies must establish their safe use in clinical practice.

## 5. Conclusions

In this study, we investigated the antibacterial properties of PTS and PTSO, two organosulfur derivatives of *Allium cepa*. These findings provide initial insights into the potential of both *Allium* species for further investigation in veterinary practice. However, in vivo trials are required to evaluate their efficacy in dogs.

## Figures and Tables

**Table 1:** Resistance percentages of *Staphyloccocus pseudintermedius* containing the *mec*A gene to *Allium* extracts and antibiotics.

	*n* (N = 30)	%
PTS	13	43.3
PTSO	13	43.3
Erythromycin	27	90.0
Ibafloxacin	25	83.3
Difloxacin	25	83.3
Enrofloxacin	25	83.3
Orbifloxacin	25	83.3
Clindamycin	24	80.0
Sulfamethoxazole Trimethoprim	24	80.0
Streptomycin Trimethoprim (TSH)	24	80.0
Spiramycin Trimethoprim (TSS)	24	80.0
Lincomycin	23	76.7
Marbofloxacin	23	76.7
Amoxicillin-clavulanic acid	17	56.7
Gentamicin	17	56.7
Pradofloxacin	15	50
Tetracycline	14	46.7
Doxycycline	14	46.7
Fusidic acid	13	43.3
Neomycin	11	36.7
Kanamycin	10	33.3
Rifampicin	9	30.0
Tobramycin	6	20.0
Chloramphenicol	3	10.0
Nitrofurantoin	0	0.0

NOTE: Sensitivity and resistance are classified using a previously established MIC cut-off, following Turnidge and Paterson (2007) [45].

**Table 2:** Comparison of percentages of *Staphylococcus psedintermedius* strains sensitive to PTS/PTSO and resistant to the other antibiotics tested.

		PTS and PTSO		
		Sensitive	Resistant	*p*-Value
Amoxicillin-clavulanic acid	Sensitive	23.3	20.0	0.454
	Resistant	33.3	23.3	
Gentamicin	Sensitive	30.0	13.3	0.388
	Resistant	26.7	30.0	
Neomycin	Sensitive	40.0	23.3	0.774
	Resistant	16.7	20.0	
Kanamycin	Sensitive	40.0	26.7	0.581
	Resistant	16.7	16.7	
Tobramycin	Sensitive	46.7	33.3	0.092
	Resistant	10.0	10.0	
Erythromycin	Sensitive	6.7	3.3	0.001
	Resistant	50.0	40.0	
Clindamycin	Sensitive	16.7	3.3	0.003
	Resistant	40.0	40.0	
Lincomycin	Sensitive	16.7	6.7	0.130
	Resistant	40.0	36.7	
Ibafloxacin	Sensitive	6.7	10.0	0.008
	Resistant	50.0	33.3	
Difloxacin	Sensitive	6.7	10.0	0.008
	Resistant	50.0	33.3	
Enrofloxacin	Sensitive	6.7	10.0	0.008
	Resistant	50.0	33.3	
Marbofloxacin	Sensitive	13.3	10.0	0.021
	Resistant	43.3	33.3	
Pradofloxacin	Sensitive	36.7	13.3	0.754
	Resistant	20.0	30.0	
Orbifloxacin	Sensitive	6.7	10.0	0.008
	Resistant	50.0	33.3	
Tetracycline	Sensitive	33.3	20.0	>0.999
	Resistant	23.3	23.3	
Doxycycline	Sensitive	33.3	20.0	>0.999
	Resistant	23.3	23.3	
Chloramphenicol	Sensitive	50.0	40.0	0.013
	Resistant	6.7	3.3	
Fusidic acid	Sensitive	33.3	23.3	>0.999
	Resistant	23.3	20	
Sulfamethoxazole/Trimethoprim	Sensitive	16.7	3.3	0.003
	Resistant	40.0	40.0	
Nitrofurantoin	Sensitive	56.7	43.3	-
	Resistant	0.0	0.0	
Rifampicin	Sensitive	43.3	26.7	0.388
	Resistant	13.3	16.7	
Streptomycin/Trimethoprim	Sensitive	16.7	3.3	0.003
	Resistant	40.0	40.0	
Spiramycin/Trimethoprim	Sensitive	16.7	3.3	0.003
	Resistant	40.0	40.0	

NOTE: Beta-lactams have been excluded due to the presence of the *mec*A gene. Colistin was not tested because it has no effect against Gram-positive bacteria. Sensitivity and resistance are classified using a previously established MIC cut-off, following Turnidge and Paterson (2007) [45]. A *p*-value < 0.05 indicates statistically significant differences between sensitivity to *Alliaceae* compounds and sensitivity/resistance to other antimicrobials.

**Table 3:** Percentages of antibiotic and PTS/PTSO resistance in ESBL-*Enterobacteriaceae*.

*Enterobacteriaceae*	*n* (N = 26)	%
PTS	12	46.2
PTSO	12	46.2
Tetracycline	25	96.2
Doxycycline	25	96.2
Ibafloxacin	23	88.5
Difloxacin	23	88.5
Enrofloxacin	22	84.6
Marbofloxacin	22	84.6
Orbifloxacin	22	84.6
Pradofloxacin	20	76.9
Colistin	17	65.4
Spiramycin/Trimethoprim	15	57.7
Gentamicin	13	50.0
Sulfamethoxazole/Trimethoprim	13	50.0
Streptomycin/Trimethoprim	13	50.0
Amoxicillin clavulanic acid	11	42.3
Nitrofurantoin	11	42.3
Chloramphenicol	10	38.5
Tobramycin	7	26.9
Neomycin	3	11.5
Kanamycin	3	11.5

NOTE: Penicillins and cephalosporins were excluded due to the presence of extended-spectrum β-lactamases (ESBLs). Erythromycin, clindamycin, fusidic acid and rifampicin were not tested against Gram-negative bacteria. Sensitivity and resistance are classified using a previously established MIC cut-off, following Turnidge and Paterson (2007) [45].

**Table 4:** Comparison of percentages of *Enterobacteriaceae* strains sensitive to PTS/PTSO and resistant to the antibiotics tested.

		PTS and PTSO		
		Sensitive	Resistant	*p*-Value
Amoxicillin-clavulanic acid	Sensitive	23.1	34.6	>0.009
	Resistant	30.8	11.5	
Gentamicin	Sensitive	26.9	23.1	>0.009
	Resistant	26.9	23.1	
Neomycin	Sensitive	46.2	42.3	0.022
	Resistant	7.7	3.8	
Kanamycin	Sensitive	46.2	42.3	0.022
	Resistant	7.7	3.8	
Tobramycin	Sensitive	30.8	42.3	0.332
	Resistant	23.1	3.8	
Ibafloxacin	Sensitive	7.7	3.8	0.003
	Resistant	46.2	42.3	
Difloxacin	Sensitive	7.7	3.8	0.003
	Resistant	46.2	42.3	
Enrofloxacin	Sensitive	7.7	7.7	0.013
	Resistant	46.2	38.5	
Marbofloxacin	Sensitive	7.7	7.7	0.013
	Resistant	46.2	38.5	
Pradofloxacin	Sensitive	11.5	11.5	0.057
	Resistant	42.3	34.6	
Orbifloxacin	Sensitive	7.7	7.7	0.013
	Resistant	46.2	38.5	
Tetracycline	Sensitive	3.8	0.0	<0.001
	Resistant	50.0	46.2	
Doxycycline	Sensitive	3.8	0.0	<0.001
	Resistant	50	46.2	
Chloramphenicol	Sensitive	30.8	30.8	0.791
	Resistant	23.1	15.4	
Sulfamethoxazole/Trimethoprim	Sensitive	30.8	19.2	>0.009
	Resistant	23.1	26.9	
Colistin	Sensitive	19.2	15.4	0.267
	Resistant	34.6	30.8	
Nitrofurantoin	Sensitive	30.8	26.9	>0.009
	Resistant	23.1	19.2	
Streptomycin/Trimethoprim	Sensitive	30.8	19.2	>0.009
	Resistant	23.1	26.9	
Spiramycin/Trimethoprim	Sensitive	26.9	15.4	0.549
	Resistant	26.9	30.8	

NOTE: Sensitivity and resistance are classified using a previously established MIC cut-off, following Turnidge and Paterson (2007) [45]. A *p*-value < 0.05 indicates statistically significant differences between sensitivity to *Alliaceae* compounds and sensitivity/resistance to other antimicrobials.

## Data Availability

Dataset available on request from the authors.

## Supplementary Materials

The following supporting information can be downloaded at: https://www.mdpi.com/article/10.3390/vetsci11010026/s1, Table S1: Susceptibility patterns of *Staphylococcus pseudintermedius* strains sensitive to PTS and PTSO; Table S2: Susceptibility patterns of *Klebsiella pneumoniae* and *Escherichia coli* strains sensitive to PTO and PTSO.
