# Supplementary material for: In Vitro Activity of Allium cepa Organosulfur Derivatives against Canine Multidrug-Resistant Strains of Staphylococcus spp. and Enterobacteriaceae
Source: Vet Sci. 2024 Jan 9;11(1):26. doi: 10.3390/vetsci11010026 (PMC10820550; doi:10.3390/vetsci11010026)
Supplement: Supplementary file 1 [file vetsci-11-00026-s001.zip › vetsci-2713020-supplementary.pdf]

Table S1: Susceptibility patterns of *Staphylococcus pseudintermedius* strains sensitive to PTS and PTSO.

Strain	ERY	CLIN	LIN	IBX	DIF	ENR	ORB	MAR	PRA	TRS	STR/TRI	SPI/TRI	AMC	DOX	TET	GEN	NEO	KA N	TOB	FUS	RIF	CHL	NIT
S. pseudintermedius	R	R	R	R	R	R	R	R	R	R	R	R	R	R	R	R	R	R	S	R	R	S	S
	R	R	R	R	R	R	R	R	S	R	R	R	R	R	R	R	R	R	R	R	R	S	S
	R	R	R	R	R	R	R	R	R	R	R	R	R	S	S	R	R	R	S	R	R	S	S
	R	R	R	R	R	R	R	R	R	R	R	R	R	R	R	S	S	S	S	R	S	S	S
	R	R	R	R	R	R	R	R	R	R	R	R	R	R	R	R	S	S	S	S	S	S	S
	R	R	R	R	R	R	R	R	R	R	R	R	S	R	R	R	S	S	S	S	S	S	S
	R	R	R	R	R	R	R	R	R	R	R	R	R	R	R	S	S	S	S	S	S	S	S
	R	R	R	R	R	R	R	R	S	R	R	R	R	S	S	S	R	R	S	S	S	S	S
	R	R	R	R	R	R	R	R	S	R	R	R	R	S	S	R	S	S	S	S	S	S	S
	R	R	R	R	R	R	R	R	S	R	R	R	S	S	S	R	S	S	S	S	S	R	S
	R	S	S	R	R	R	R	S	S	R	R	R	R	R	R	S	S	S	R	R	S	S	S
	R	R	R	R	R	R	R	R	S	R	R	R	S	S	S	S	S	S	S	S	S	S	S
	R	S	S	R	R	R	R	R	S	S	S	S	S	S	S	R	S	S	S	S	S	S	S
	R	S	S	R	R	R	R	R	S	S	S	S	S	S	S	S	S	S	S	S	S	S	S
	R	R	R	S	S	S	S	S	S	S	S	S	S	S	S	S	S	S	S	S	S	R	S
	S	S	S	R	R	R	R	S	S	S	S	S	R	S	S	S	R	R	R	R	R	S	S

Abbreviations: R:resistant, S:sensitive; ERY: erythromycin, CLIN: clindamycin, LIN: lincomycin, IBX: Ibafloxacin, DIF: Difloxacin, ENR: Enrofloxacin, ORB: Orbifloxacin, MAR: Marbofloxacin, PRA: Pradofloxacin, TRS: Sulfamethoxazole/Trimethoprim, STR/TRI: Streptomycin/Trimethoprim, SPI/TRI: Spiramycin/Trimethoprim, AMC: Amoxicillin-clavulanic acid, DOX: Doxycycline, TET: Tetracycline, GEN: Gentamicin, NEO: Neomycin, KAN: Kanamycin, TOB: Tobramycin, FUS: Fusidic acid, RIF: Rifampicin, CHL: Chloramphenicol, NIT: Nitrofurantoin

Table S2: Susceptibility patterns of *Klebsiella pneumoniae* and *Escherichia coli* strains sensitive to PTO and PTSO.

Strains	DOX	TET	IBX	DFX	ENR	MAR	PRA	ORB	COL	AMC	TRS	CHL	SPI/TRI	NIT	STR/TRI	GEN	TOB	NEO	KAN
K. pneumoniae	R	R	R	R	R	R	R	R	R	R	R	R	R	R	R	R	R	R	R
K. pneumoniae	R	R	R	R	R	R	R	R	R	R	R	R	R	R	R	R	R	R	R
K. pneumoniae	R	R	R	R	R	R	R	R	R	R	R	R	R	R	R	R	R	S	S
K. pneumoniae	R	R	R	R	R	R	R	R	S	R	R	R	R	R	R	R	R	S	S
K. pneumoniae	R	R	R	R	R	R	R	R	S	S	S	R	S	R	S	R	S	S	S
K. pneumoniae	R	R	R	R	R	R	S	R	R	R	R	S	R	S	R	R	S	S	S
E. coli	R	R	R	R	R	R	R	R	R	R	R	S	R	R	R	R	R	S	S
E. coli	R	R	R	R	R	R	R	R	S	R	S	S	S	S	S	S	R	S	S
E. coli	R	R	R	R	R	R	R	R	R	S	S	R	S	S	S	S	S	S	S
E. coli	R	R	R	R	R	R	R	R	R	S	S	S	S	S	S	S	S	S	S
E. coli	R	R	R	R	R	R	R	R	R	S	S	S	S	S	S	S	S	S	S
E. coli	R	R	R	R	R	R	R	R	S	S	S	S	S	S	S	S	S	S	S
E. coli	R	R	S	S	S	S	S	S	R	R	S	S	S	S	S	S	S	S	S
E. coli	S	S	S	S	S	S	S	S	S	S	R	S	S	S	S	S	S	S	S

Abbreviations: R:resistant, S:sensitive; IBX: Ibafloxacin, DIF: Difloxacin, ENR: Enrofloxacin, ORB: Orbifloxacin, MAR: Marbofloxacin, PRA: Pradofloxacin, COL: colistine,TRS: Sulfamethoxazole/Trimethoprim, SPI/TRI: Spiramycin/Trimethoprim, STR/TRI: Streptomycin/Trimethoprim, AMC: Amoxicillin-clavulanic acid, DOX: Doxycycline, TET: Tetracycline, GEN: Gentamicin, NEO: Neomycin, KAN: Kanamycin, TOB: Tobramycin, CHL: Chloramphenicol, NIT: Nitrofurantoin
